# Technical comparison of MinIon and Illumina technologies for genotyping Chikungunya virus in clinical samples

**DOI:** 10.1186/s43141-023-00536-3

**Published:** 2023-08-29

**Authors:** Leandro Menezes de Souza, Isabelle Dias de Oliveira, Flávia Cristina Silva Sales, Antonio Charlys da Costa, Karoline Rodrigues Campos, Adriano Abbud, Juliana Mariotti Guerra, Cinthya dos Santos Cirqueira Borges, Carlos Pires Fernandes Júnior Takahashi, Leonardo José Tadeu de Araújo

**Affiliations:** 1 Centro de Patologia, Instituto Adolfo Lutz, Sao Paulo, Brazil; 2 Programa de Pós Graduação em Ciências da Saúde do Instituto de Assistência Médica ao Servidor Público Estadual – IAMSPE, Sao Paulo, Brazil; 3 Departamento de Moléstias Infecciosas e Parasitárias, Instituto de Medicina Tropical, Faculdade de Medicina da Universidade de São Paulo, Sao Paulo, Brazil; 4 Centro de Respostas Rápidas, Instituto Adolfo Lutz, Sao Paulo, Brazil; 5 State Health Secretary of São Paulo, São Paulo, Brazil

**Keywords:** Next-generation sequencing, Metric MinION and Illumina, Chikungunya virus genotype

## Abstract

New-generation sequencing (NGS) techniques have brought the opportunity for genomic monitoring of several microorganisms potentially relevant to public health. The establishment of different methods with different mechanisms provides a wide choice, taking into account several aspects. With that in mind, the present aim of the study was to compare basic genomic sequencing metrics that could potentially impact genotyping by nanopores from Oxford Nanopore Technologies and by synthesis from Illumina in clinical samples positive for Chikungunya (CHIKV). Among the metrics studied, running time, read production, and *Q* score were better represented in Illumina sequencing, while the MinIOn platform showed better response time and greater diversity of generated files. That said, it was possible to establish differences between the studied metrics in addition to verifying that the distinctions in the methods did not impact the identification of the CHIKV virus genotype.

## Background

Genomic surveillance techniques are constantly used to monitor various aspects associated with the existence of agents that cause emerging/re-emerging diseases [13]. Since the introduction of next-generation sequencing platforms, such as Roche's pyrosequencing in 2005 [31] and Illumina/Solexa’s sequencing by synthesis in 2007 [32], it has become necessary to make the platforms less difficult to use and more affordable. Given this situation, Oxford Nanopore Technologies launched MinIon in 2014, the first commercial sequencer that was easy to use, even in the most basic laboratory settings, and able to perform direct sequencing on clinical and environmental samples [20].

This suggestion for a more affordable portable sequencer opened the door to the possibility of expanding the monitoring and response to viral outbreaks like the Ebola virus [29], Poxvirus [8], Dengue virus [17], Zika virus [28], and more recently SARS-CoV-2 [12].

The technical aspects of genomic monitoring sequencing may be viewed and researched; hence, the sequencing platform should be carefully selected based on the application. Because of its mobility, third-generation sequencing technologies like as MinION give real-time monitoring of the produced data, allowing investigations to run concurrently with the sequencing and giving enhanced information on viral circulation [16].

Between epidemiological weeks (EW) 01-13 of 2021, the State of São Paulo documented 1079 confirmed cases of Chikungunya virus (CHIKV) infection [1]. As its transmission is intricately tied to the presence of *Aedes aegypti* and *A. albopictus* mosquitoes [27], this alphavirus, belonging to the Togaviridae family of single-stranded RNA viruses [34], has garnered substantial attention in Public Health. The goal of this work, in the context of genomic surveillance, was to undertake a technical analysis connecting and comparing metrics generated from MinIon and Illumina sequencing of positive CHIKV samples.

## Materials and methods

### Sample selection and viral nucleic acid extraction

The study included eight frozen serum samples that were previously screened by RT-qPCR at the Instituto Adolfo Lutz (IAL) for CHIKV detection. The following parameters were used as inclusion criteria: RT-qPCR detection, threshold cycle value < 30 (to maximize genome coverage of clinical samples) and adequate storage conditions. RNA extraction was performed with the Extracta Kit®-RNA and viral DNA (MVXA-P096 FAST) using the Extracta 96 equipment and following the supplier’s protocol.

### RT-qPCR for CHIKV

The RT-qPCR assay was based on the SYBR system using the SYBR Green Quantitative RT-qPCR Kit (SIGMA-ALDRICH®, QR0100) on the ABI PRISM 7000 system. The probes and cycling conditions were standardized according to [15]. The Ct value represented the cycle by which the fluorescence of a sample increased to a level greater than the background fluorescence in the amplification cycle.

### Chikungunya genome amplification and amplicon pools

RNA amplification was followed by reverse transcription and cDNA synthesis using the Superscript IV VILO cDNA Synthesis Kit (Invitrogen, USA). Then, the production of amplicons was constituted from the multiplex reaction using the Q5® High-Fidelity DNA Polymerase Kit (New England BioLabs®, USA), where 92 pairs of primers were used in the construction of 4 distinct pools, which were used in the amplification of the samples in quadruplicates. The CHIKV primers for this protocol were designed based on the ZiBRA Project (https://www.zibraproject.org/) and generate ~ 400 nt overlapping amplicons [28]. The generated amplicons were cleaned up using magnetic beads (Agencourt® AMPure XP-Beckman Coulter, USA), then divided in two and used in the construction of both the Oxford Nanopore and Illumina libraries. The amplicons were measured using fluorimetry with the dsDNA® HS Assay Kit (Thermo Fisher Scientific) on the Qubit 3.0 (Life Technologies, USA). Two amplicon pools that did not obtain satisfactory concentrations were discarded.

### Library construction for nanopore sequencing

The library contained 120 ng of final input. In this context, all samples were normalized to 20 ng per sample in a final volume of 20 µL. The amplicons were repaired, associated with barcodes and adapters, and included in the preparation of the library using the following kits: NEBnext Ultra II End Repair/dA-tailing (New England Biolabs, USA), EXP-NBD104 (Oxford Nanopore Technologies, UK) linked to the NEBnext Ultra II Ligation Module Ligase Kit (New England Biolabs, USA), and SQK-LSK109 (Oxford Nanopore Technologies, UK). The library application took place in a previously primed R9.4.1 flow cell (Oxford Nanopore Technologies, UK), which was inserted in the MinION equipment (MinKNOW 1.15.1). The sequencing data was available in about 24 h.

### Library construction for sequencing by synthesis

The library construction followed the protocol provided by the manufacturer for the Illumina DNA Prep Kit (illumina, San Diego, USA). The pool amplicons input was 20 ng per sample, following the steps of tagmenting genomic DNA, post-tagmenting cleanup, amplifying tagged DNA, cleaning up libraries, and pooled libraries. The MiSeq Reagent Kit V3 sequencing system was used to obtain paired end readings with an average size of 2 × 75 bp.

### Generation of consensus sequences and genotype identification

After the completion of the sequencing runs, fast5 files were filtered, polished, and demultiplexed using Guppy basecaller 2.2.7 (Oxford Nanopore Technologies, UK). The reads were mapped to the reference genome (GENBANK, MT526904) thus obtaining consensus files according to the genome coverage obtained (Geneious, v.2021.2.2). Nanopolish polished the consensus; therefore, it did not carry regions of the genome with less than 25X coverage. For Illumina, FastQ files were obtained through Base Space, and the consensus was determined using the Geneious software, considering readings with 55X sensitivity.

For both platforms, the negative control with null coverage confirmed that there was no contamination in the sequenced libraries. For this study, the genotype was determined using the online Genome Detective (https://www.genomedetective.com/).

### Ethical statement

This study was conducted with human serum samples suspected of acute febrile infection, which were screened and approved for use under the CAAE 96138818.0.0000.0059. The sequencing experiments were conducted at the Laboratório Estratégico do Instituto Adolfo Lutz (LEIAL), and bioinformatics analyses were carried out at the Núcleo de Patologia Quantitativa (NPQ). The generated sequences were deposited in Genbank under the following accession numbers: OL898707, OL898700, OL898693, OL898686, OL898679, and OL898713.

## Results

### Workflows

**Fig. 1:**
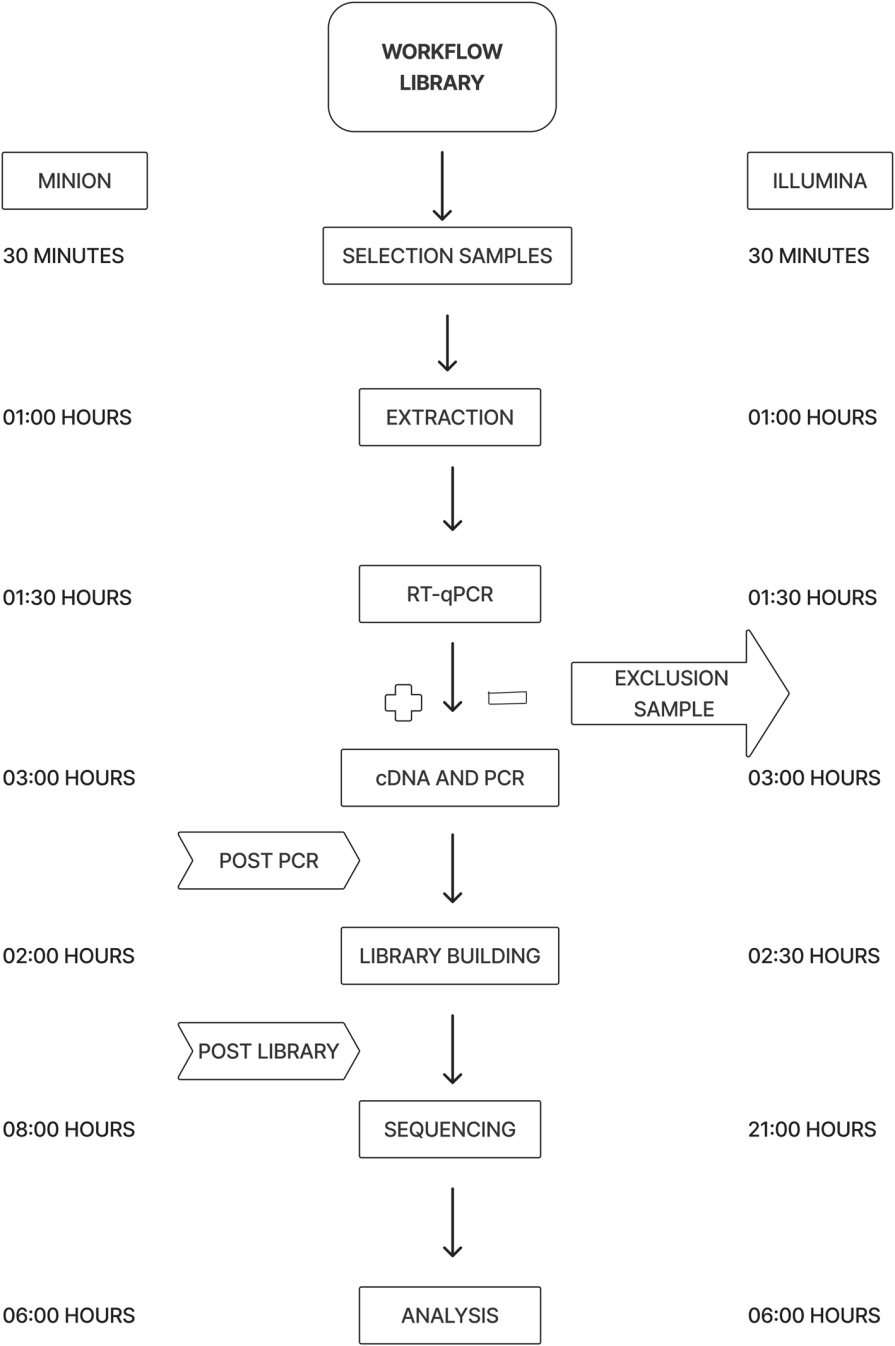
Workflow for library preparation

The sequencing workflow for both platforms was divided into pre-library, library construction, and sequencing. From sample quality control through sequencing itself, this flow needed a minimum of 18 h of benchwork for MinIon libraries and 20 h for Illumina libraries as detailed in (Fig. 1), and might be increased depending on the number of samples.

The preparation of the sample pools that converged the entire CHIKV viral genome required from 6 to 7 h of work, from selection and extraction to cDNA and PCR, where there was no difference in the time spent between the methods (Fig. 1). The cost per sample until multiplex PCR amplification was around US$ 50 when using reagents that were already standardized for this purpose. The MinIon platform provided a good experience in terms of cost because, on average, the values do not deviate from the standard, with a total cost of approximately US$ 60 until the sequencing itself. However, its greatest cost-per-sample benefit is the availability of a technique that assures that libraries with a high number of samples minimize their costs, in addition to having means to reuse some consumables.

The preparation of the sample pools that converged the entire CHIKV viral genome required from 6 to 7 h of work, from selection and extraction to cDNA and PCR, where there was no difference in the time spent between the methods (Fig. 1). The cost per sample until multiplex PCR amplification was around US$ 50 when using reagents that were already standardized for this purpose. In terms of cost, the MinIon platform provided a good experience because, on average, the values do not deviate from the standard, with a total cost of around US$ 60 until the sequencing itself. However, its greatest cost-per-sample benefit is the availability of a technique that assures that libraries with a high number of samples minimize their costs, in addition to having means to reuse some consumables.

There were significant differences in post-sequencing processing in terms of base calling, demultiplexing, adapter removal, and low-quality reads. The methods used are shown in (Table 1 and 2), and the Geneious Prime software was used for both the alignment and the assembly of the consensus sequence. Because Illumina's methodology is based on paired-end reads, the Illumina platform produced two times as much raw data as MinION sequencing.

**Table 1:** Factors that distinguish MinION sequencing from Illumina sequencing

Parameter	MInIon	Illumina
Output file	Fast5/ FastQ/FASTA	FastQ
Pre library runtime	6 h	
Library runtime	hands on: 50 min	hands on: 1 h
	hands off: 1 h 10 min	hands off: 1 h 30 min
Run time	8 h	21 h
Initial analysis	6 h	
Max. data size (per run)	1 Gb	3.3 Gb

**Table 2:** Consensus sequence construction steps by methodology

Step	Illumina	MinION
Base calling	Bcl2FASTQ (Åslin et al. 2018) [4]	Guppy [36]
Demultiplexing		
Removing low-quality reads	Cutadapt	
Removing adapters and primers	Trimmomatic [5]	Pipelines ARTIC (Artic Network) [3]
Alignment and consensus	Geneious Prime v. 2021.2.2	

The Illumina platform in general provided better standardized results between samples in the sequencing and analysis step, where its results generally surpass those of the Minion platform. When the number of samples is small, the cost per sequencing run or sample rises dramatically. The Minion platform, on the other hand, allows you to sequence a single sample without incurring additional costs.

### Genome detection

Initially, the FASTQ files from the MinION and Illumina sequencing runs were run through the FASTQC reports tool to assess the read quality. The standard *Q* score for MinION was 21, and for Illumina it was 37, allowing us to say that the readings produced by MinION had a risk of error per base of 1:100, or 99% accuracy in its reading, whereas the error per base rounded up for Illumina was 1:10.000, or about 99.9% accuracy (Table 3). Data such as genotype, coverage, and identity in percentage of nucleotides were extracted using the Genomic Detective platform (Table 3). We observed that all samples belonged to the East-Central-South-African (ECSA) lineage clade.

**Table 3:** Genomics features provided by MinIon and Illumina sequencing

Genome CHIKV	Methods	Coverage %	Reads	NT identify %	AA identify %	Concordance %
*OL898679*	MinION	97.8	136,863	95.5	97.8	94.4
	Illumina	98.7	544,777	96.5	98.2	93.2
*OL898686*	MinION	97.8	129,813	95.7	97.9	94.7
	Illumina	98.7	340,485	96.5	98.2	93.2
*OL898693*	MinION	97.8	108,215	95.6	97.9	94.8
	Illumina	98.7	544,777	96.5	98.2	93.2
*OL898700*	MinION	97.8	131,092	94.5	97.3	93.1
	Illumina	98.7	544,777	96.5	98.2	93.2
*OL898707*	MinION	97.8	163,899	93.7	96.5	91.0
	Illumina	98.7	544,777	96.5	98.2	93.2
*OL898713*	MinION	97.8	156,405	96.7	98.2	96.1
	Illumina	98.7	544,777	96.5	98.1	93.2

Coverage: rate percentage given to the generated sequence in detriment to the reference genome

Reads: nucleotide sequence taken as part sequenced genome

NT identify %: rate in percentage of correspondencen of the nucleotides that make up the generated sequence

AA identify %: rate in percentage of correspondencen of the aminoacids that make up the generated sequence

Concordance: Metric related to the level of equality between the generated sequence and the reference sequence

NCBI Reference Sequence: NC_004162.2

When examining the amount of reads generated, it was clear that the set of samples sequenced by MinION exhibited a pattern of equivalent diversity between samples, with the number of reads being considerably lower compared to the reads produced by the Illumina platform. These discrepancies had little effect on the genomic coverage achieved, but had a greater impact on the homogeneity of other measures, such as nucleotide identification rate.

**Fig. 2:**
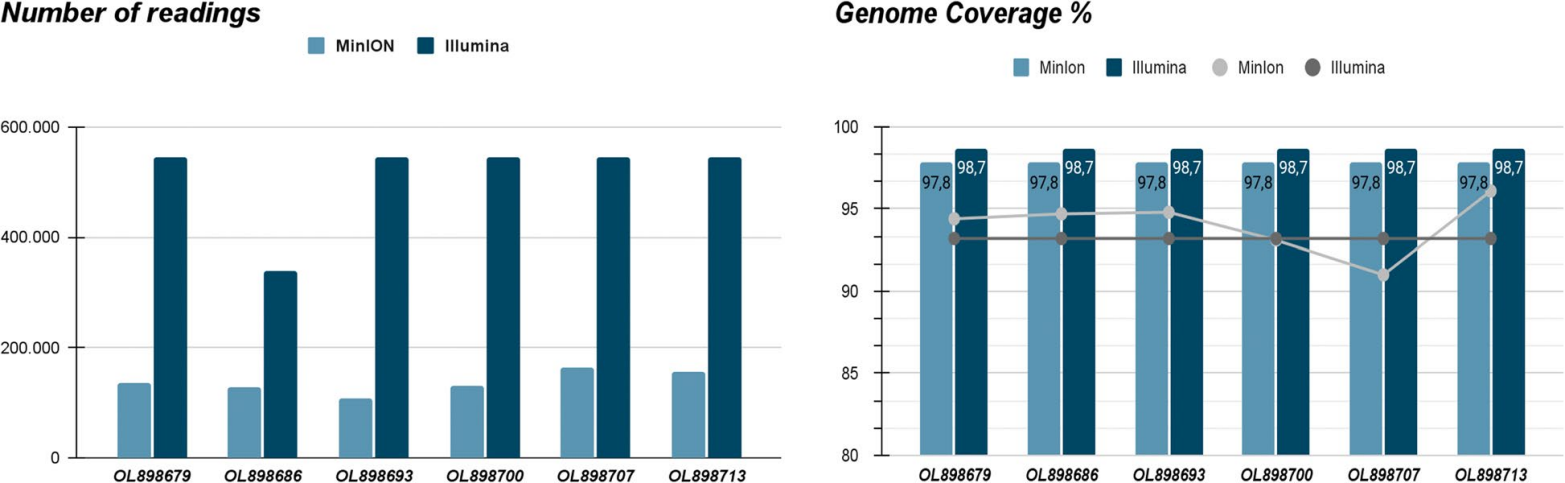
MinION and Illumina number of reads (**A**) and genomic coverage and concordance genomic per sample (**B**)

The Illumina platform produced more uniform genomic measurements across samples, indicating superior standardization of library construction and avoiding sequencing competition. Although we have not achieved 100% coverage, we can see that the nucleotide similarity and identification rate is very accurate compared to the reference sequence. The collected mean, standard deviation and coefficient of variation data suggest a small and unapparent variation in the number of reads obtained from Minion and Illumina sequencing, which may have been impacted in some way by the limited number of sequenced samples, given that both platforms had low dispersion when seen by the coefficient of variation, presenting 13.6% for MinION and 16.3% for Illumina. The mean and standard deviation for the respective platforms were: 133,977; 18.3 and 510,723; 83.4. When evaluating the number of average reads produced, the MinION platform produced an absolute value greater than the Illumina platform (Fig. 2); however, there was a greater dispersion between samples in the MinION group.

## Discussion

In reality, the use of MiniON and Illumina sequencing has differences and technical peculiarities, but as previous research has noted, demonstrating the subtleties of the approaches in a comparison is a challenge [19]. Illumina sequencing technology was previously utilized as a gold standard approach to demonstrate the efficacy of standardization in a research study building a Minion sequencing process utilizing the multiplex PCR scheme [28]. Depending on the study, each approach is well-suited, and each has benefits and disadvantages that must be addressed when the goal is determined [10].

Given that the results gathered here vary slightly between investigations, it is obvious that, although having equal capacity, the selection of kits, supplies, and analysis methods is critical. Several variables are discovered that might impact the metrics and results achieved; yet, both methods guarantee to identify genotypes with high accuracy and consensus sequences with good agreement [35]. The comparative need between methodologies took place in parts to resolve the doubt that although the MInION methodology generally appears to bring sequences with a *Q* score below 20, its adaptability and immediacy in the time taken to obtain an answer shows that this methodology can offer a better option against the quick response [26].

The coverage depth ratio used by the method influences the production of sizable readings; while the MinION platform generally considers readings with 25 × coverage, the Illumina platform considers readings with possibly greater depth of coverage. Due to the lower genomic depth score of the MinION platform, reads with a lower number of copies per genomic region of the reference sequence were admitted as equivalent readings, increasing the absolute number of reads per sequence. On the Illumina platform, reads produced above 55 × were considered equivalent, but the Illumina library was built with a pool of samples that were enriched with a multiplex PCR before sequencing, probably explaining the not-so-high difference between the production of reads per sample [25, 33].

Currently, using the nanopore sequencing approach from Oxford Nanopore Technologies, it is feasible to sequence microorganisms in the environment in real time, allowing this procedure to be replicated in difficult-to-access locations without relying on sophisticated laboratory buildings [12]. However, because of its benefits, the availability of analytic tools, and the accuracy of the results provided, Illumina's MIseq platform is the most widely utilized approach today.

Costs, infrastructure, collaborator network, input availability, and raw data analysis capability often restrict genomic surveillance tactics, which are directly tied to the benefits of each sequencing method [25]: Sanger to analyze mutations in small conserved genes [14], MinION for rapid detection of viral outbreaks [7] and Illumina for high precision and viral subtyping [21]. That said, there are several studies that compared the usual power of each method against a specific need. Russell et al. (2018) [30] compared the number of reads and the average size between sequences produced by MinION and Illumina technologies in pools of captured Culicidae in the field; Vries et al. (2022) [11] used as a comparative basis the genomic coverage between sequences corresponding to the viral glycoprotein hemagglutinin of the influenza virus using MinION and Illumina; and King et al. (2020) [18] considered the MinION and Ion Torrent comparison viable, since the possibility of sample multiplexing and real-time read selection made the MInIon platform prone to use in rapid diagnostics.

What separates the sequencing generations are usually the principles of the methods. llumina platform for sequencing is called the second-generation method of next-generation sequencers, based on the creation of short-read sequences based on the synthesis of the concomitant sequences [23]. To differentiate from second-generation techniques, third-generation MinION sequencing is based on portability and the creation of long-read libraries based on target enrichment reactions through the PCR reaction [6]. Despite the similarities in terms of time spent, seen here, in the assembly of sequencing libraries, there are more unique points that differ in the methods, in addition to each one presenting unique means of analysis, which are standardized according to the principles of each method. Both approaches provide significant changes in the development of the library as a result of the methods’ principles, and the availability of inputs is of similar availability, giving speed of reactions to viral outbreaks of the same parameter [22]. However, the Minion technology is known to have higher *Q* score rates of reads than the other sequencing generations. This is due to the requirement to build amplicons by PCR prior to sequencing, as well as the method of transforming electrics in the pore into base, where the electrical interruption sensitivity is minimal, allowing conversion errors [9].

Previous studies with a comparative focus have corroborated the findings shown here, since both the MinION and the Illumina methodologies exert influence on genomic findings that do not require analysis of point mutations and intra-hopeter interactions [24]. Also, initial analysis in the identification of outbreaks has the same investigative power, emphasizing that the use of methodologies such as the MinION platform allows real-time analysis without the need to finish the sequencing run. Thus, there are numerous studies that lead us to believe that, for initial analyses, both platforms have equivalent power [2]. When establishing a specific objective, it is always necessary to evaluate the use, knowing that both may differ in terms of analysis.

## Conclusion

It is possible to infer that, despite their technical differences, both approaches fulfilled the same objective of determining the genotype of the CHIKV virus, demonstrating that choosing the best strategy is reliant on the intended goal.

## Data Availability

The datasets generated and/or analyzed during the current study are available in the [GENBANK] repository, [https://www.ncbi.nlm.nih.gov/nucleotide/].

## Abbreviations

CAAE: Certificate of Presentation of Ethical Appreciation
CHIKV: Chikungunya virus
ECSA: East-Central-South-African
IAL: Institute Adolfo Lutz
LEIAL: Laboratório Estratégico do Instituto Adolfo Lutz
NGS: Next-generation sequencing
NPQ: Núcleo de Patologia Quantitativa
